# Accurate spatiotemporal mapping of drug overdose deaths by machine learning of drug-related web-searches

**DOI:** 10.1371/journal.pone.0243622

**Published:** 2020-12-07

**Authors:** David S. Campo, Joseph W. Gussler, Amanda Sue, Pavel Skums, Yury Khudyakov

**Affiliations:** 1 Division of Viral Hepatitis, National Center for HIV/AIDS, Viral Hepatitis, STD, and TB Prevention, Centers for Disease Control and Prevention, Atlanta, GA, United States of America; 2 Georgia State University, Atlanta, Georgia, United States of America; University of Cincinnati College of Medicine, UNITED STATES

## Abstract

Persons who inject drugs (PWID) are at increased risk for overdose death (ODD), infections with HIV, hepatitis B (HBV) and hepatitis C virus (HCV), and noninfectious health conditions. Spatiotemporal identification of PWID communities is essential for developing efficient and cost-effective public health interventions for reducing morbidity and mortality associated with injection-drug use (IDU). Reported ODDs are a strong indicator of the extent of IDU in different geographic regions. However, ODD quantification can take time, with delays in ODD reporting occurring due to a range of factors including death investigation and drug testing. This delayed ODD reporting may affect efficient early interventions for infectious diseases. We present a novel model, Dynamic Overdose Vulnerability Estimator (DOVE), for assessment and spatiotemporal mapping of ODDs in different U.S. jurisdictions. Using Google^®^ Web-search volumes (i.e., the fraction of all searches that include certain words), we identified a strong association between the reported ODD rates and drug-related search terms for 2004–2017. A machine learning model (Extremely Random Forest) was developed to produce yearly ODD estimates at state and county levels, as well as monthly estimates at state level. Regarding the total number of ODDs per year, DOVE’s error was only 3.52% (Median Absolute Error, MAE) in the United States for 2005–2017. DOVE estimated 66,463 ODDs out of the reported 70,237 (94.48%) during 2017. For that year, the MAE of the individual ODD rates was 4.43%, 7.34%, and 12.75% among yearly estimates for states, yearly estimates for counties, and monthly estimates for states, respectively. These results indicate suitability of the DOVE ODD estimates for dynamic IDU assessment in most states, which may alert for possible increased morbidity and mortality associated with IDU. ODD estimates produced by DOVE offer an opportunity for a spatiotemporal ODD mapping. Timely identification of potential mortality trends among PWID might assist in developing efficient ODD prevention and HBV, HCV, and HIV infection elimination programs by targeting public health interventions to the most vulnerable PWID communities.

## Introduction

Injection drug use (IDU) is associated with an increase in overdose deaths (ODDs), HIV and hepatitis C virus (HCV) infections, and other noninfectious health conditions [1–3]. From 2010 to 2015, the number of HCV new infections jumped by 294%, with particularly sharp increases among states hardest hit by the opioid crisis [4]. These IDU-related infections are associated with a high cost of health care and high mortality, presenting a substantial public health problem. Spatiotemporal identification of PWID communities and assessment of the health conditions associated with IDU might assist in identifying hard-to-reach populations most vulnerable to infections with bloodborne pathogens, which require targeted public health interventions to reduce health disparity among these populations.

Reported ODD from different geographic regions is a fundamental metric for estimating key factors associated with PWID (e.g., community size or IDU risk) and for assisting in developing and implementing interventions. Overdose is the most frequent cause of death among PWID, with annual mortality rates for PWID being 14–17 times greater than for their non-drug using peers [2]. Although timeline of drug overdose death reporting has improved in recent years [5], ODD quantification can take time, with delays in ODD reporting occurring due to a range of factors including death investigation and drug testing. Hence, CDC Wonder has 11–23 months delays in final data availability. Although 12-month ending provisional counts (97–99% complete) of ODD are now available after only a 6-month lag [5], a monthly estimate may enable a more dynamic ODD surveillance to aid in identifying regions that may be at risk for increased morbidity and mortality associated with IDU.

Web-searches are a powerful method for monitoring intention and interest in a topic, which has been termed *predicting the present*, and demonstrate their utility with monitoring economic indicators [6]. Web-searches were first used successfully in epidemiology for predicting incidence of influenza-like illnesses [7, 8], giving rise to the practice of what is now referred to as digital epidemiology [9–13]. With regard to ODD, it was found that increases in online posts about synthetic opioids precede increases in synthetic opioid death rates [14]. In addition, Young et al. [15] studied the association of opioid-related Web-searches with future heroin-related admissions to emergency departments. Their model explained 72% of the variance in heroin-related emergency visits across nine metropolitan areas in the United States, indicating potential applicability of digital epidemiology in assessing IDU risks [15].

We hypothesize that observed ODD trends could be used as a link between the activity of the PWID community and the easily accessible and recent Web-searches data. The accuracy of ODD predictions can then be used to prune the data and thus find the most important keywords. Thus, we implemented a novel tool, the Dynamic Overdose Vulnerability Estimator (DOVE), for surveillance and assessment of ODD trends by using Web-searches. Applying this tool could provide an opportunity for the detection of changes in ODDs in vulnerable communities, so that public health investigations can be targeted to areas with potential increases in ODDs and IDU-related infections.

## Methods

### Data

#### Observed ODD data

For each state for 2004–2017, we gathered the number of yearly and monthly deaths caused by drug poisonings (overdose), including ICD-10 underlying cause of death codes X40–X44, X60–X64, X85, and Y10-Y14 [16]. For each U.S. county in the same period, we gathered number of yearly and monthly deaths in the same way. The crude rates per 100,000 persons were used, with both monthly and yearly estimates using the yearly population. We did not use age-adjusted rates in any of the analyses. Data were obtained from the Centers for Disease Control and Prevention’s National Center for Health Statistics, Underlying Cause of Death 1999–2017 on CDC WONDER Online Database, released in December 2018. Data were obtained from the Multiple Cause of Death Files, 1999–2017, as compiled from data provided by the 57 vital statistics jurisdictions through the Vital Statistics Cooperative Program [17]. For many counties (e.g., 68.73% for 2017), the exact ODD number is suppressed because of privacy concerns when the number of deaths is <10. We assigned estimates for these counties on the basis of the procedure outlined by Tiwari et al. [18], who demonstrated that effects of suppression can be largely overcome by using the known total suppressed count and partitioning these counts according to county population. Although we could have requested access to the suppressed data, we wanted this research to be strictly based on publicly available data, either from the CDC wonder side or google, so our work can be better replicated and validated by others. Because of the large portion of suppressed data for the monthly county estimates (95.16% for 2017), we excluded the monthly county estimates from further analysis.

#### Web-search data

We obtained from Google Trends™ (Google, Inc., Mountain View, California) the query rates of 80 drug-related keywords (Table 1) in each state for each month and year from January 1, 2004, to December 31, 2018. The rate for each query is calculated as the total query volume for the search term in question within a state divided by the total number of queries in the state during the period examined [6]. Google states that the these rates have the following characteristics (internally defined by Google, Inc): (i) the queries are broad-matched; therefore, such queries as *drug overdose* were counted in the calculation of the rates for *overdose*; (ii) rates are computed from a subset of all data collected using a sampling method, and the results, therefore, varied slightly from day to day; and (iii) due to privacy considerations, only queries with a certain minimum volume are reported. Because of restrictions from Google, Inc. on access to county-level Web-search rates, the model was trained by using only the state-level Web-search rates.

**Table 1:** List of web-search keywords and their association with ODD rate in 2017.

Keywords (A–L)	Correlation	MultiSURF Rank	Keywords (M–Z)	Correlation	MultiSURF Rank
adderall	0.2646	29	meperidine	-0.0006	59
addict	0.5365	53	methadone	0.6470	7
addiction	0.2050	45	morphabond	0.1764	36
alprazolam	-0.0375	72	morphine	0.2045	21
amphetamine	0.1739	61	murder eight	0.0000	42
apadaz	-0.1013	51	naloxone	0.6690	10
arymo	0.1747	36	narcan	0.8318	1
bath salts	-0.0911	39	narcotic	0.1806	64
benzodiazepine	0.0755	66	norco	-0.2641	70
buprenorphine	0.5564	43	opana	0.3360	33
china girl	0.1244	19	opiate	0.5191	16
china white	0.2758	30	opioid	0.7508	9
cocaine	0.4039	48	overdose	0.6644	8
codeine	-0.1581	50	oxy	-0.1159	22
cody	-0.1759	49	oxycodone	0.2561	67
demerol	-0.1422	34	oxycontin	0.1905	52
depression	0.1930	25	oxymorphone	0.3806	68
diazepam	-0.0604	20	pain	0.4410	12
dilaudid	0.2608	75	perc	0.6644	5
dope	0.0463	27	percocet	0.4655	17
dope sick	0.2587	63	percocet 30	0.5393	35
dose	0.1249	56	pill	0.2318	69
drug dealer	0.5724	11	purple drank	-0.3003	58
duragesic	0.0498	80	rehab	0.2690	13
dying	0.1937	47	ritalin	0.1632	62
embeda	-0.0044	40	roxybond	0.0432	41
exalgo	0.1949	73	shooting up	0.3032	55
fentanyl	0.5394	6	sober	0.0305	74
fenty	0.2558	23	suboxone	0.7092	4
goodfella	0.3349	31	subutex	0.5695	14
hep c	0.4867	44	subutex vs suboxone	-0.1918	18
heroin	0.7707	2	targiniq	0.0863	36
hydrocodone	-0.3915	15	tattoo	0.0256	28
hydromorphone	0.0299	71	track marks	0.3112	79
hysingla	-0.0446	57	tramadol	-0.0356	32
iv drug	0.3892	65	veteran	0.0936	77
king ivory	-0.1454	54	vicodin	0.3052	76
laxative	0.1752	78	vikes	-0.2672	26
			vivitrol	0.7505	3
			withdrawal	0.6469	24
			xtampza	0.2972	60
			zohydro	-0.1299	46

### Analysis

#### Individual features

To measure the association strength of a keyword with the ODD rates, we calculated the individual Pearson correlation across all 51 regions (50 states and the District of Columbia) for each target year.

#### Machine learning overview

The entire dataset includes all available years (e.g., 2004–2017), with 80 keywords and 1 response variable (ODD data). The following is an overview of the machine learning procedure.

- We split the dataset into a validation dataset (last known year, 2017), and a working dataset (all previous year, 2004–2016).
- For each target year in the working dataset (e.g., 2005), we calculated the prediction accuracy (measured as Median Absolute Error percentage [MAE]) of a model trained using only previous years (e.g., 2004) and tested on the target year.
- This was repeated with a variety of parameter combinations (feature selection levels, machine learning methods, number of previous years used, levels of temporal information, and levels of spatial information), for a total of 2048 combinations for each of the 12 target years of the working dataset. The average of each combination over the 12 evaluated years was calculated.
- This way, parameter tuning allowed us to select not the best parameters for the entire training dataset, but rather parameters that performed well predicting unseen data over a long period of time for each test year.
- Finally, the best value for each parameter was chosen and the model with these parameters was applied to the validation dataset. This dataset has never been seen by the model before and thus will allow us to estimate its true predictive power.

#### Feature selection

Machine learning is improved by feature selection methods that can function in noisy problems and detect complex patterns of association between variables. Here we applied the MultiSURF method to choose a subset of the keywords, which is a Relief-Based Algorithm that yields the most reliable feature selection performance across a wide range of problem types [19]. For each model, we calculated the MultiSURF score of each keyword variable and ranked them. For each model, 15 levels of numbers of variables were tested, ranging from 5 variables to 75 variables in steps of 5.

#### Machine learning method

Multiple machine learning methods were tested, including Ordinary least-squares, Bayesian ridge regression, Lasso, Adaptive Boosting, k-nearest neighbors, Decision trees, random forest, and Extremely Random Forest (ERF) [20, 21]. ERF demonstrated the best average results over all years evaluated. We used the scikit-learn [22] Python implementation of ERF with default sci-kit parameters.

#### Number of previous years used

We studied models with two levels of previous years used: (i) only the previous year (e.g., if the target year is 2006, training only with 2005) or (ii) all available years (e.g., if the target year is 2006, training with both 2005 and 2004).

#### Temporal information

We tested two levels of temporal information: (i) without any extra information and (ii) with a single added variable containing the lagged observed ODD rate, which is obtained from the last available value for that particular area, in a similar manner to Kandula and Shaman [13]. Thus, besides the keyword rates, this level includes the information about the ODD rate in each studied region from the previous year.

#### Spatial information

We tested four levels of spatial information: (i) without any extra information; (ii) with a simple dummy variable for each state, where a datapoint has a 1 if its state is the one for the variable; (iii) with a spatial neighborhood structure, as suggested by Sandahl [23], where there is a variable for each state and a datapoint has a 1 if its state is the same as the variable or if it is a geographic neighbor of the state; and (iv) with a population weighted spatial neighborhood structure, where there is a variable for each state and a datapoint has a 1 if its state is the same as the variable, or if it is a geographic neighbor of the state it gets a number equal to the neighboring state population divided by the sum of all neighboring populations.

#### County-level estimation

We calculated county estimates as follows: (i) For each county, we calculate percentage of the overall observed state ODD counts that belong to each county. As there are several years’ worth of data, the final percentage was the expected value for the new year based on the trend obtained by linear regression. (ii) ODD rates are predicted for each state using Web-searches. Then, the predicted rate is used to estimate the number of ODD counts based on the state population size. (iii) The predicted state ODD counts are then partitioned among counties according to their final percentage. (iv) Finally, these county ODD counts are used to calculate the county rate by dividing by the counts the county population size.

## Results

### Correlation of individual web-search terms with ODD rates

**Fig 1:**
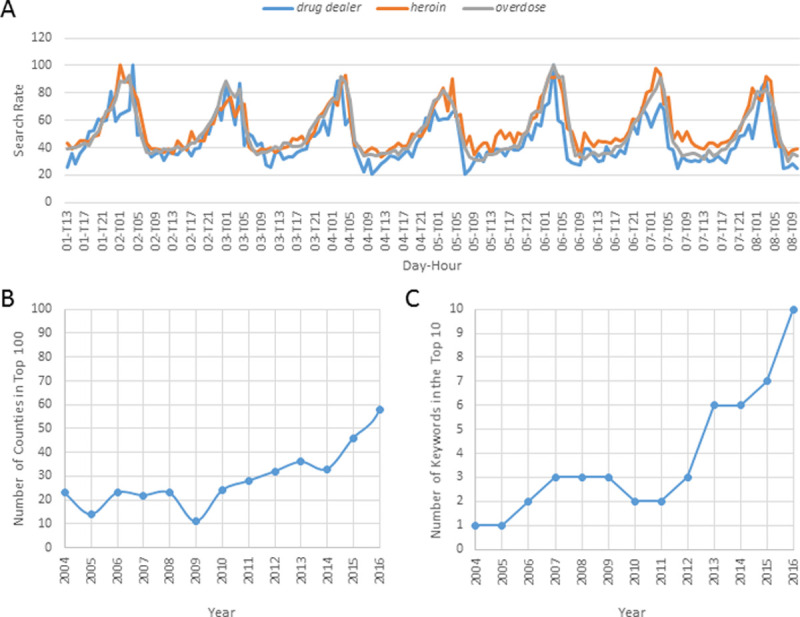
Dynamic nature of opioid and search rates. (A) Search Rates of 3 Drug-Related Terms for October 1, 2019–October 8, 2019; (B) For Each Year, Number of Counties in the Top 100 (by reported overdose death [ODD] Rate) That Are Also in the Top 100 Counties in 2017; (C) For Each Year, Number of Keywords in the Top 10 (By Correlation with reported ODD Rates) That Are Also in the Top 10 Keywords Identified for 2017.

Our interest in Web-search terms started with the observation that the search rates of certain drug-related terms (*drug dealer*, *heroin and overdose*) revealed daily peaks 1–3 hours after midnight, especially on weekends (Fig 1A). Afterwards, we found by using https://www.google.com/trends/correlate that most keywords associated with the state ODD rates (from the year 2015) were drug-related terms such as *overdose*, *narcan*, and *suboxone*. Due to this finding, we proceeded to measure the association between the reported yearly ODD rates and drug-related keywords for each year during the 2004–2017 period. A list of 80 web-search terms included a mixture of: (i) common drug names, (ii) new slang terms and (iii) terms related to high risk populations or behaviors (e.g. “veteran”), and (iv) terms with high correlation with the state rates of 2015 using Google correlate.

Among 80 search terms evaluated (Table 1), 24 were highly correlated with the reported ODD rate during 2017 (Pearson correlation, *P* < .01). The 10 most correlated terms were *narcan* (r = 0.8318; *P* < .0001), *heroin* (r = 0.7707; *P* < .0001), *opioid* (r = 0.7508; *P* < .0001), *vivitrol* (r = 0.7505; *P* < .0001), *suboxone* (r = 0.7092; *P* < .0001), *naloxone* (r = 0.6690; *P* < .0001), *perc* (r = 0.6644; *P* < .0001), *overdose* (r = 0.6644; *P* < .0001), *methadone* (r = 0.6470; *P* < .0001), and *withdrawal* (r = 0.6469; *P* < .0001). All correlation measures shown refer to the state level.

### Variation of web-search terms over time and among states

**Fig 2:**
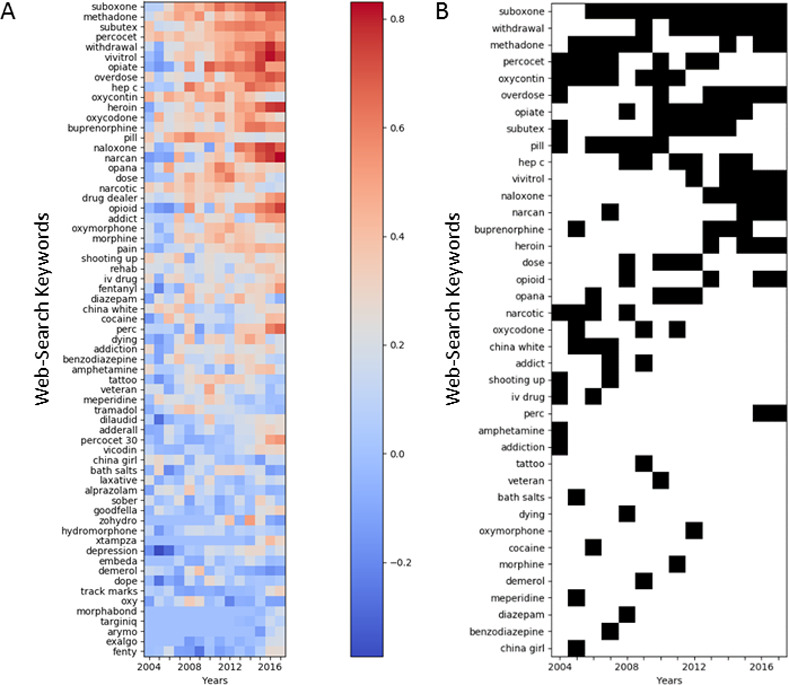
Changes across time. (A) Correlation of 80 Keywords with observed Overdose Death (ODD) Rates for Each Year, 2004–2017, Keywords Are Sorted from the Highest to the Lowest Average Correlation Across All Years; (B) Keywords in the Top 10 (Shown in Black) by Correlation with observed ODD Rates for Each Year, 2004–2017.

Out of the 100 counties with the highest observed ODDs during 2017, only 58 were among the top 100 during 2016 and only 11 during 2009. These changes indicate the highly dynamic nature of the ODD rate variation (Fig 1B). This dynamic nature can also be observed among the top 10 keywords correlated with ODDs during 2017, where only a few were also in the top 10 during previous years (Fig 1C). Correlation of individual keywords with ODD rates over all states in a particular year varied over time (Fig 2A). The considerable change of the top 10 keywords between consecutive years (Fig 2B) indicates a highly dynamic association between Web-search terms and ODDs. With limited exceptions (e.g., *suboxone*, *methadone*, or *withdrawal*), where association with ODDs constantly increases over time (consistent and strong association with ODDs during the previous 2–3 years), the association of the majority of keywords fluctuated between consecutive years (e.g., *opana*, *fentanyl*, or *diazepam*).

**Fig 3:**
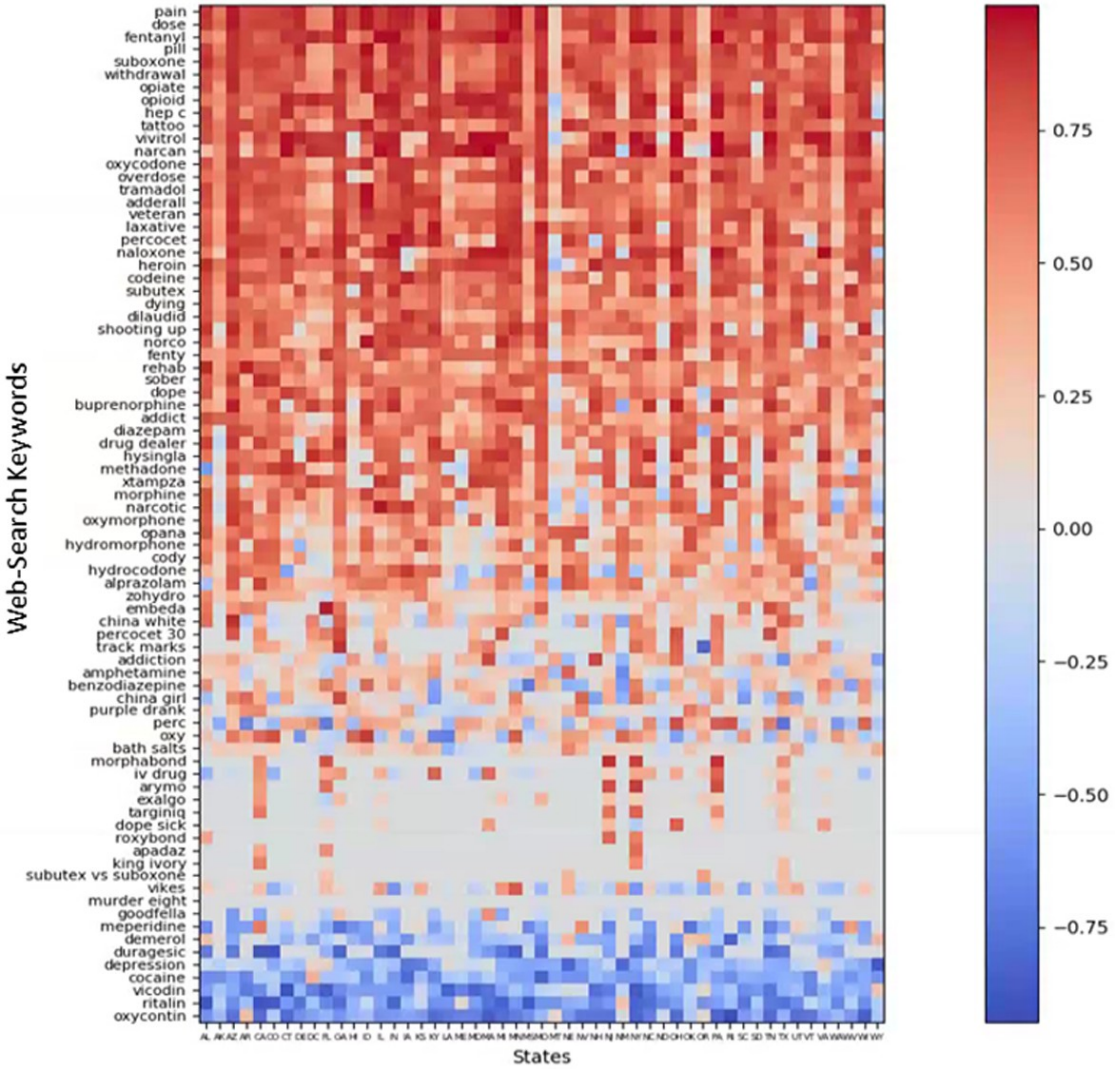
Correlation between web-search volumes by using individual keywords and yearly observed overdose death rates by states during 2004–2017.

A noticeable variation of the association of ODDs with use of different Web-search keywords was observed among states (Fig 3). Some of the terms among the 20 most correlated with ODDs (Fig 3) (e.g., *narcan* or *vivitrol*) did not show high correlation with ODDs for certain states (e.g., Arkansas, Hawaii, or Oregon). Correlation of other terms (e.g., *perc* or *benzodiazepine*) varied broadly from negative to positive values among states (Fig 3). Besides individual terms, overall correlation with ODDs varied among states, with certain states (e.g., Arizona, California, Georgia, Ohio, and Pennsylvania) having a high overall correlation, whereas others (e.g., Montana, North Dakota, Oregon, Washington, and Wyoming) had low overall correlations.

### Machine learning with the working dataset (2005–2016)

**Fig 4:**
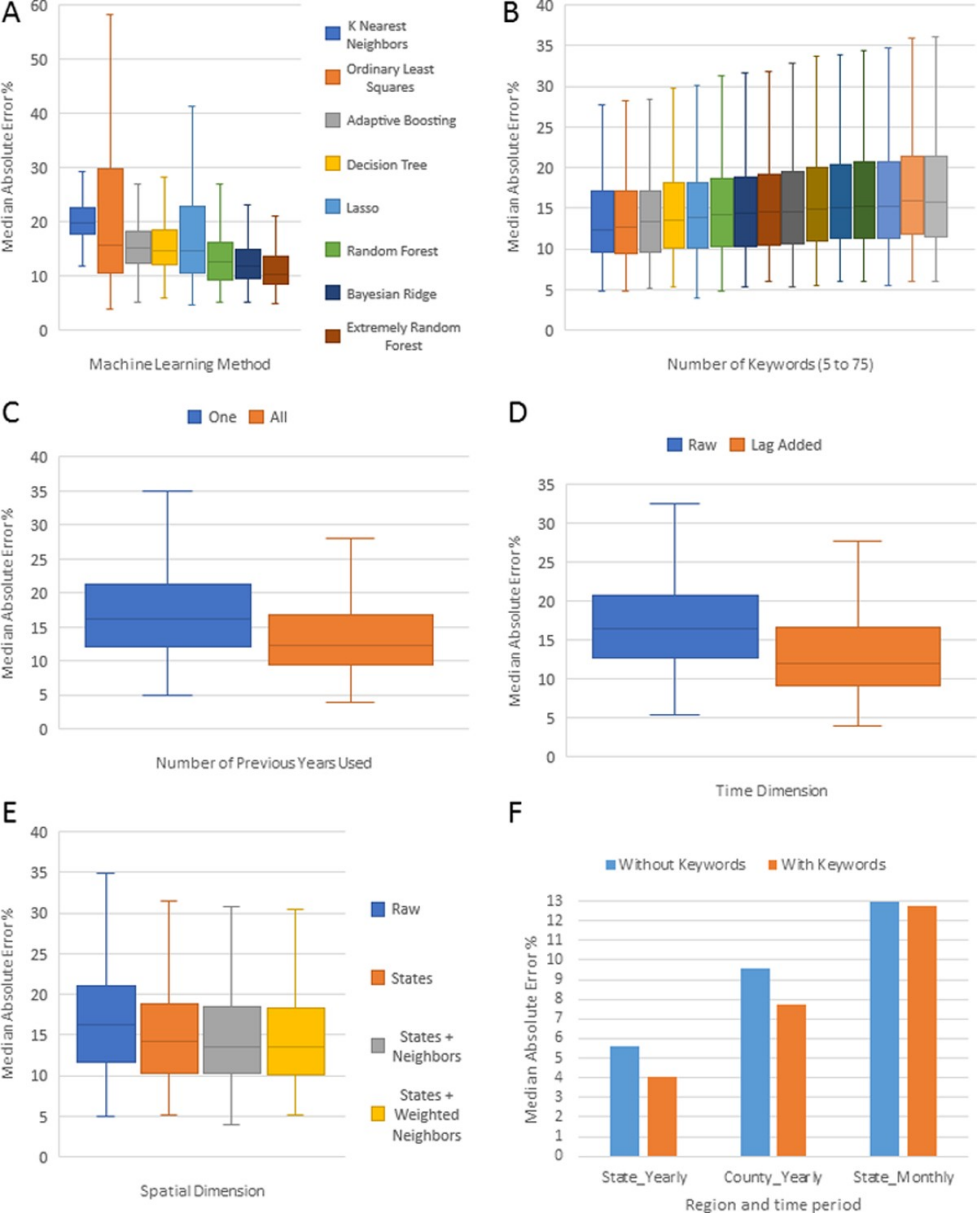
Machine learning performance in the working dataset. (A) Machine Learning Methods; (B) Number of top keywords used (from 5 to 75, in steps of 5); (C) Number of previous Years Used; (D) Inclusion of lag observed ODD rate; (E) Inclusion of spatial variables; (F) Comparison of the final model’s performance in 2017 with and without Web-search Terms.

Given that we have several years’ worth of data, we can search for a model that performs well under several years with dynamic changing conditions. We split the dataset into a validation dataset (last known year, 2017), and a working dataset (2004–2016). For each target year in the working dataset, we calculated the MAE of a model trained using only previous years (e.g., 2004 and 2005) and tested on the target year (e.g. 2006). This was repeated with a variety of parameter combinations for each of the 12 target years of the working dataset. The average of each combination over the 12 values was calculated and the best value for each parameter chosen:

- The best method was ERF (Fig 4A), both having the lowest MAE across all parameter combinations and the lowest variation across years. A close second was Bayesian Ridge Regression. ERF fits several randomized decision trees on various sub-samples of the dataset and uses averaging to improve the predictive accuracy and control over-fitting.
- The best number of top keywords (by MultiSURF score) was 5 (Fig 4B).
- The MAE of models using all available previous years was lower than the MAE of models using only one immediately previous year (Fig 4C).
- The MAE of models including a lag variable of the ODD rate was lower than the MAE of models lacking this addition (Fig 4D).
- The MAE of models including spatial information was lower than the MAE of models lacking spatial information. The best MAE occurred with the addition of a spatial neighborhood structure, where there is a variable for each state and a datapoint has a 1 if its state is the same as the variable name or if it is a geographic neighbor of the state (Fig 4E).

**Fig 5:**
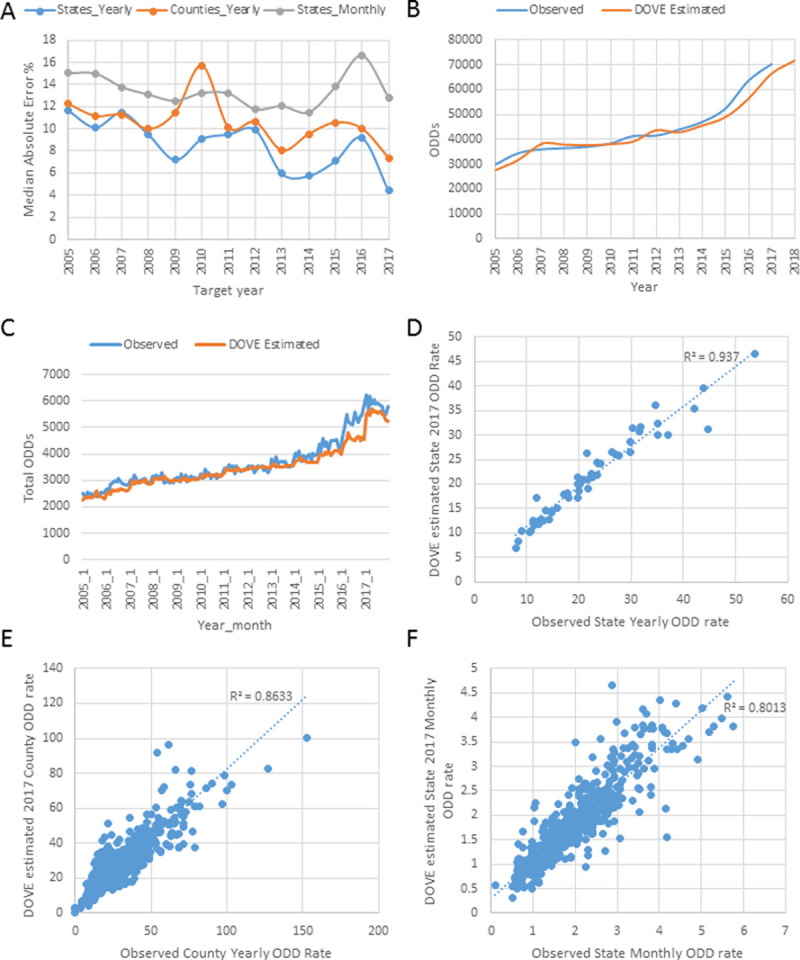
Dynamic Overdose Vulnerability Estimator (DOVE) performance. (A) Median Absolute Error % of the Final Model for Each Target Year; (B) Total number of observed and predicted yearly ODDs; (C) Total number of observed and predicted Monthly ODDs; (D) Predicted versus Observed State 2017 Overdose Death (ODD) Rates; (E) Predicted versus Observed County 2017 ODD Rates; (F) Predicted versus Observed State Monthly ODD Rate in 2017.

Over the entire working dataset, the model with this particular combination of parameters (ERF, 5 top keywords, all previous years, previous year ODD rate, added spatial neighborhood structure) showed a MAE of 9.32%, 10.59%, and 13.19% among yearly estimates for states, yearly estimates for counties, and monthly estimates for states, respectively (Fig 5A). The model estimated a total of 487,161 ODDs out of the reported 501,946 (97.05%) during this time period (Fig 5B). As these results come from our training dataset, the more relevant results are for 2017 as shown below.

### DOVE assessment for 2017

The validation dataset (2017) has not been used for building the final model. This set was tested only to make the final evaluation of the model performance. The following 5 keywords were identified as the most relevant for assessing ODDs during the period 2004–2016 due to their high MultiSURF score: *narcan*, *heroin*, *vivitrol*, *suboxone*, and *perc* (Table 1).

DOVE estimated 66,463 ODDs out of the reported 70 237 (94.48%) during 2017. The median error of the individual ODD rates was 4.43%, 7.34%, and 12.75% among yearly estimates for states, yearly estimates for counties, and monthly estimates for states, respectively (Fig 5A). These results were better than the models lacking web-search keywords (using only the lag rate and the spatial neighborhood structure) (Fig 4F). Error of yearly DOVE estimates for 2017 varied among states from 0.04% in Utah to 44.78% in Wyoming. Estimates for all regions, except the District of Columbia, Vermont, and Wyoming, were made with less than 20% error, whereas the most accurate estimates (<1% error) were obtained for Florida, Kansas, Maine, New York, Oregon, Tennessee, and Utah.

The DOVE estimates for counties were as follows: (i) Out of the top 100 U.S. counties by ODDs, 91 were also in the top 100 identified by DOVE; (ii) 75 of the reported top 100 U.S. counties by ODD rates were also in the top 100 identified by DOVE; (iii) When comparing the ranks of all counties, the correlation between observed and predicted ODD rates was very high (Spearman r = 0.9569, P < 0.001).

### Dynamic spatiotemporal ODD mapping

**Fig 6:**
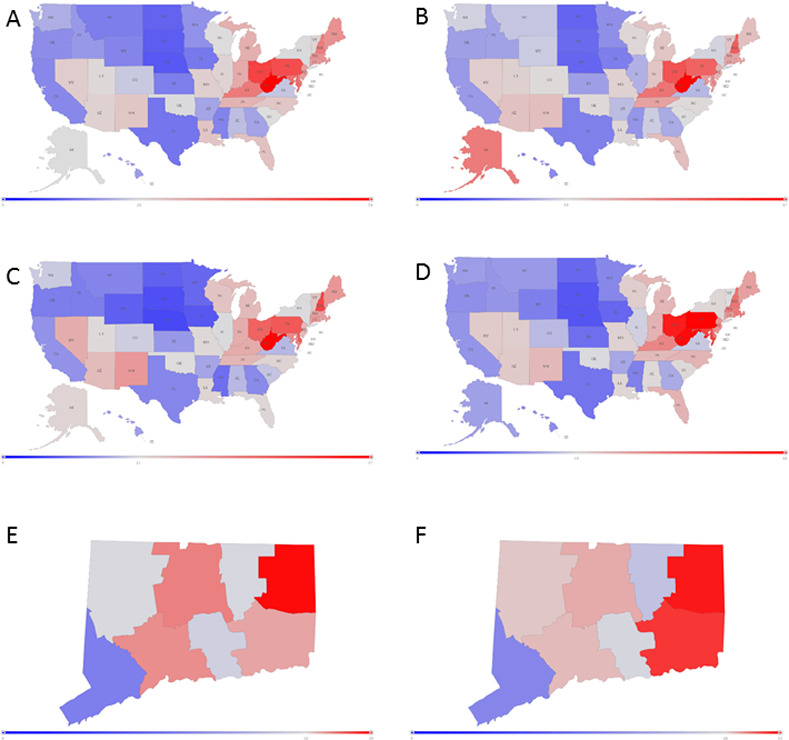
US maps generated by the Dynamic Overdose Vulnerability Estimator (DOVE). The color corresponds to the ODD rate, from low (blue) to high(red). (A) Observed 2017 Overdose Death (ODD) Yearly Rates by State; (B) DOVE Estimated 2017 ODD Yearly Rates by State; (C) Observed July 2017 ODD Rates by State; (D) DOVE Estimated July 2017 ODD Rates by State; (E) Observed County 2017 ODD Yearly Rates in the State of Connecticut; DOVE Estimated County 2017 ODD Yearly Rates in the State of Connecticut.

DOVE can capture changes dynamically, whereas the lag in ODD reporting by using hard data is 11–23 months. DOVE estimated 71,622 ODDs occurred during 2018, which was 5.94% higher than the reported true value (n = 67,367). DOVE assessments can be obtained almost instantly for the past month or year at the state level and for the past year at the county level. Fig 6 displays examples of maps generated by DOVE, comparing the reported versus estimated ODDs by states and counties for the state of Connecticut. This state showed the lowest error while having none of its counties suppressed, thus the similarity of observed and predicted is separated from the uncertainty on the method to assign rates to suppressed counties.

We investigated if the error was associated with population size, Internet usage penetration (as of 2017) [24], or fraction of population in urban areas (as of 2010) [24]. The error in our estimates is not correlated with the percentage of the population in each state that uses the Internet (Pearson correlation = 0.0076; *P* = 0.9580), nor with population size (-0.2359; 0.0956), nor with the fraction of population in urban areas (-0.0494; 0.7309).

## Discussion

With some limitations presented below, DOVE provides a simple dynamic spatiotemporal mapping of ODD at the level of U.S. states and counties by using Web-searches. Internet-derived information has been applied recently to epidemiologic investigations of different infectious and noninfectious diseases, giving rise to the practice of what is now referred to as digital epidemiology [9–13]. Google Trends is most frequently used for assessing epidemiologic trends from Web-searches [25, 26]. Although examples of successful use of digital epidemiology in different epidemiologic settings exist [15, 27, 28], reliable application of the web-search information to public health is still under investigation [25, 26]. Recently, Kandula and Shaman [13] reappraised the utility of Google Flu Trends, showing how the initial failures of the Google Flu Trends could have been avoided by using different techniques.

To a substantial degree, accuracy of assessments relies on strength of linkage of an epidemiologic variable chosen for evaluation with Web-search volumes. Here, we used ODDs because we expected them to generate a robust and specific response among Internet users. Indeed, we identified a strong association between selected drug-related search terms and reported ODD, resulting in the 95.02% accuracy of the DOVE assessment. DOVE’s accuracy varied among jurisdictions and across time, producing epidemiologically relevant estimates with yearly median errors of 11.64% and 14.23% among states and counties, respectively. Considering a variable association of search terms with ODD among states and counties, the model might require specific term adjustments to improve performance in jurisdictions with lower DOVE accuracy. However, overall, DOVE’s performance improved with each year of observation, which can be explained simply by an increased amount of data (as more previous years are available) or by the increased correlation of individual variables with ODD across time. Although the reasons for this increase are unclear, the contribution of increased Internet usage, resulting in a greater volume of data is a reasonable explanation. If this is true, the model will continue improving with time, with the Internet providing more data and more specific terms.

The availability of sensitive diagnostic tests and highly effective therapy capable of achieving sustained virologic response in >95% of patients have made HCV infection elimination technically feasible in the United States and globally [29]. Given that PWID are at highest risk for HCV transmission, making them a high priority for treatment [1], spatiotemporal identification of PWID communities and assessment of the health conditions associated with IDU provides potential means of identifying vulnerable populations needed for targeted interventions. ODD estimates are vital for assessment of IDU risk and associated HIV, HBV, and HCV infections as well as an array of noninfectious health conditions. ODDs have been used to establish county-level vulnerability scores for acquisition of HIV infection [30]. However, considering the time and effort needed to assess ODD, the estimates are outdated, which limits their timely application among such dynamic groups as PWID. In comparison with reported ODDs obtained by using hard data, estimates made by DOVE, while less exact, can be obtained more quickly, thus facilitating its effective application in identifying areas that may warrant further investigation. However, it must be considered that our county estimates are ultimately based on a partitioning of the state estimate based on previous behavior. This means that within a state, DOVE estimates are not better than estimates based on the trend of previous years, for instance obtained by forecasting models. However, when counties are compared at the national level, DOVE estimates become useful as they still incorporate the updated web-based estimate and thus changes in ranking at the national level can identify areas where ODD rates may have recently increased.

**Fig 7:**
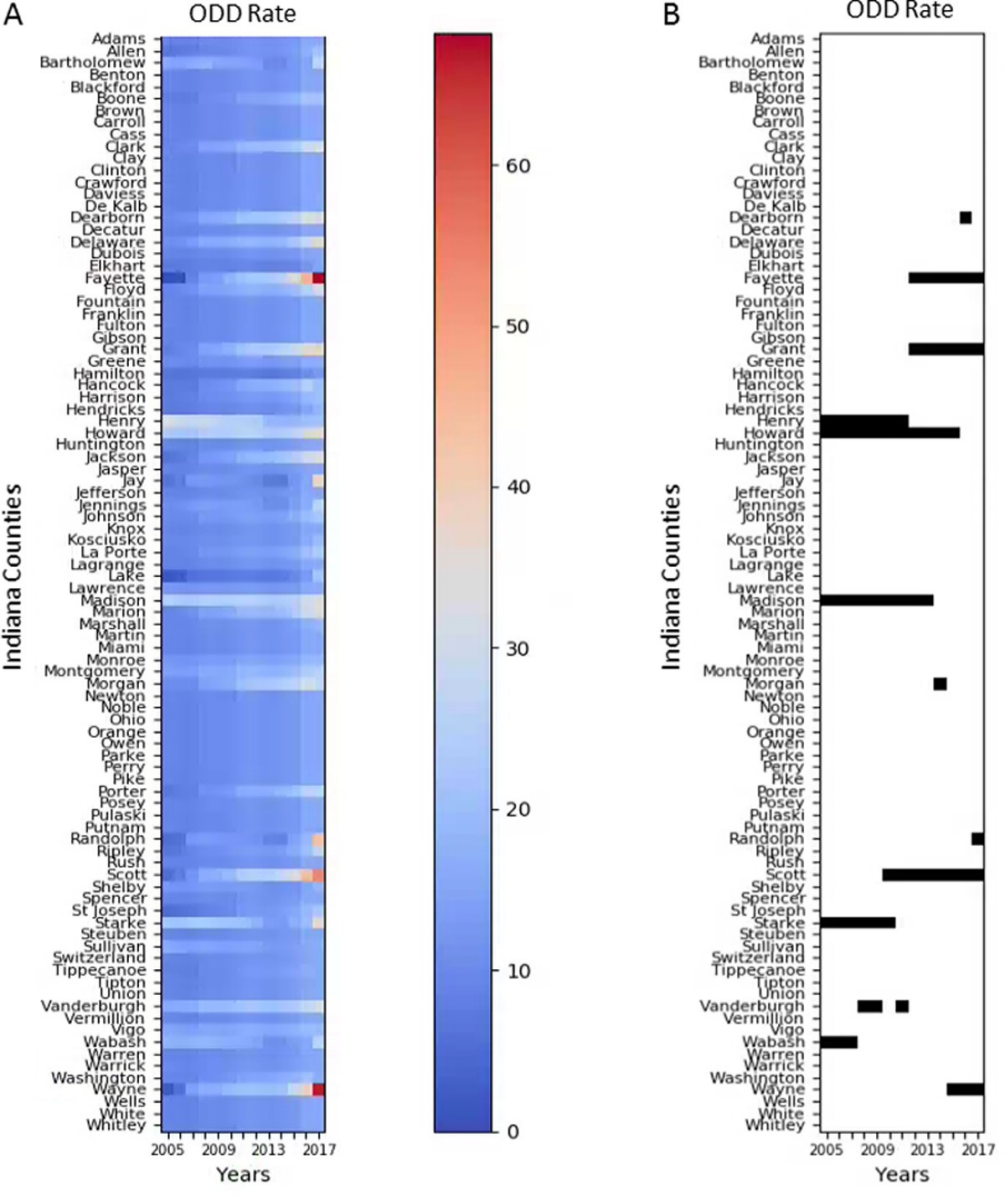
DOVE estimates for Indiana counties during 2005–2017. (A) Estimated Overdose Death (ODD) Rate; (B) Top Indiana 5 Counties by ODD Rate Estimated by DOVE for Each Year.

A major HIV and HCV infection outbreak that occurred in Scott County, Indiana [31–33] lends support to the need for a more dynamic monitoring among vulnerable populations. During November 2014–November 2015, the Indiana State Department of Health recorded a cluster of 11 HIV infections in a small rural community in Scott County. Further investigation led to detection of 181 HIV-positive patients linked to injection use of oxymorphone [31–33]. Genetic analyses revealed a longstanding and continued HCV transmission within this affected community, and a dense and dynamic network of HCV transmission among PWID that enabled extensive HIV transmission [33]. A detailed analysis of this outbreak indicates that transmission began in 2011, underwent a considerable increase in mid-2014, and decreased after public health interventions [34]. In retrospect, Scott County was one of the top 5 Indiana counties for ODD rates in 2010, with the highest county ODD rate in both 2011 and 2012, as well as the 12th highest U.S. county rate for 2011 and 13th highest for 2012. Although this was the period just before the county experienced a substantial HIV and HCV infection outbreak associated with PWID, the final data for each of these years were released 2–3 years later (Fig 7). This episode highlights an instance where DOVE could have better informed program decision-making.

Application of technologies like DOVE, when capable of detecting dynamic changes in ODD rates, could assist public health efforts, especially after DOVE is enhanced with actual web-based county rates. Reported ODDs can be used as a strong metric of changes in the activity and size of the PWID community. However, surveillance systems have delays in data availability for months, highlighting the need for timely and comprehensive ODD surveillance. The recorded ODD values are highly accurate but not current. Although high accuracy of estimates is crucial, sensitivity to dynamic ODD variations is key for effective application of estimates in public health interventions among PWID. Technologies like DOVE might serve as a virtual advance warning of potential rapid changes in vulnerability, which, for example, might have helped in developing timely interventions for reducing HIV and HCV infection transmission in Scott County. DOVE is now available in GHOST (Global Hepatitis Outbreak and Surveillance Technology) [35]. GHOST is a cloud-based platform where only registered Public Health Users have access to a suit of bioinformatic tools to aid in Hepatitis surveillance and outbreak detection. As part of GHOST we have several “X-labs”, where new beta-release tools are tested and troubleshooted before wide release. DOVE has been implemented as an X-lab into GHOST, currently providing state-yearly, state-monthly and county-yearly “vulnerability scores”, which are ranks obtained using our predicted ODD rates. The purpose of DOVE is to work as a smoke alarm and find places with a growing PWID community which can be vulnerable to the spread of viral infections.

A noticeable variation exists in association of ODDs with Web-search keywords among jurisdictions, indicating a locally specific usage of the terms. Thus, keywords must be continuously updated to keep the model relevant. Although one of the advantages of our model is the ease of keywords addition and modification without any additional cost, we are currently exploring the automation of this selection process by using natural language processing of drug-related reports. This offers an opportunity for adjusting messages of the targeted advertisements based on rapidly changing local jargon to improve communication and prevention interventions.

This study has several limitations: (i) Owing to the dynamic nature of keyword usage in Web-searches, the model presented here does not capture current ODD trends equally well for all states and needs to be continuously updated to account for the rapid changes in the usage of specific search terms. Thus, we must be proactive with the update of keywords, otherwise the model will stop being useful. Although one of the advantages of our model is that it is very easy to add keywords that may be important without any cost, we are currently exploring the automation of this selection process by using natural language processing of drug-related reports; (ii) Extrapolation of the county suppressed ODD rates used in this study does not allow for accurate assessment of current ODD rates for such counties, making these results preliminary. Research is warranted for further improvement of the model based on using data from all counties including counties with suppressed data. In addition, the complete data will allow the use of age-adjusted rates, which may improve accuracy; (iii) Owing to privacy concerns, data for county specific Web-searches are not readily available, which significantly affects accuracy of ODD assessments, especially monthly assessments, at the county level. This is another potential venue for further improvement of the model; (iv) Although monthly estimates at the state level can help detect shifting trends and sudden changes in ODD, these estimates can only guide where further investigation for targeting resources to the appropriate communities might be warranted; (v) Web searches can be sensitive to the overall burden of drug use in a community despite reduction in fatal overdoses as the community becomes more familiar with new drugs and overdose treatments, which may potentially explain an overestimate of the ODD numbers for 2018 by DOVE (predicted slowdown vs observed decline); (vi) We have not found a satisfactory explanation for the wide variation in error rates among states, which suggests that finding a measurable variable that accounts for this variation could substantially improve the model; (vii) It must be noted that given the delays in data availability (with 11–23 months for final data), the last available known rate used in our models will not always be from the previous year (as modelled here) but sometimes from the year before that. Thus, accuracy of predictions may differ in a real-world setting, although even models without any lag variable performed well. Moreover, 12-month ending provisional counts (97–99% complete) of ODD are now available after only a 6-month lag [5] and could be used to bring the lagged rates closer to their current value.

DOVE was designed as an alert system, in the same way a smoke alarm detects fire. We aim to detect major changes in vulnerability to acquisition of HIV, HBV and HCV infections and noninfectious health conditions associated with IDU, by using ODD to calibrate the model. Such major changes leading to large disease outbreaks are unlikely to occur in counties with suppressed ODD data within few years because of small PWID population, whereas substantial changes in vulnerability among large PWID communities may have a significant effect on acquisition of infections as was observed in Scott County. However, further improvement of the theoretical framework used in this study may help develop models for the accurate, dynamic estimation of ODD without at least some of the aforementioned limitations.

## Conclusions

The model presented here is a novel surveillance tool for spatiotemporal mapping of ODD rates. In the absence of other information, these ODD estimates could be used to monitor major changes in potential vulnerability to IDU-related acquisition of HIV, HBV, and HCV infections.
